# Association Between Visceral Adipose Tissue and Depression Risk With Both Observational and Genetic Evidence

**DOI:** 10.1002/brb3.71392

**Published:** 2026-04-23

**Authors:** Linxiao Gao, Haoyu Fang, Yanhe Liu, Wen Luo, Lingxiang Xu

**Affiliations:** ^1^ Division of Liver Surgery Department of General Surgery West China Hospital of Sichuan University Chengdu China; ^2^ Department of Hepatobiliary Surgery The Second Affiliated Hospital of Chongqing Medical University Chongqing China

**Keywords:** depression, joint association, physical activity, visceral adipose tissue

## Abstract

**Background:**

Previous studies have demonstrated a link between visceral obesity and depression. Nevertheless, there is a lack of sufficient studies examining the relationship between visceral adipose tissue volume (VATV) and depression, particularly concerning the combined influence of physical activity on this connection.

**Methods:**

The study analyzed data from the National Health and Nutrition Examination Survey cycles spanning 2011–2018. A total of 7460 individuals aged between 20 and 59 years were included. VATV was evaluated using either dual‐energy x‐ray absorptiometry or magnetic resonance imaging. Multivariate logistic regression models were applied to investigate the relationship between VATV and depression. Mendelian randomization analysis was carried out to examine potential causal relationships.

**Results:**

In the fully adjusted multivariate logistic regression model, a significant positive association was found between VATV and depression (OR, 1.04; 95% CI, 1.01–1.07). Lower VATV levels combined with effective physical activity significantly reduced depression prevalence. Mendelian randomization analysis identified a causal relationship between VATV and depression.

**Conclusions:**

Our study highlights a positive association between visceral fat and depression. Furthermore, participants who regularly engage in physical activity and have low VATV levels are more strongly associated with a reduced prevalence of depression. Importantly, the causal association between them was confirmed. These findings underscore the potential of managing visceral fat to prevent depression.

## Graphical abstract

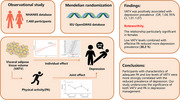

**Abbreviations**
AIC: Akaike's information criterion
CI: confidence intervals
DXA: dual‐energy x‐ray absorptiometry
GWAS: genome‐wide association studies
HPA: hypothalamic‐pituitary‐adrenal.
IEU: integrative epidemiology unit
IVs: instrumental variables
IVW: inverse‐variance weighted
MR: Mendelian randomization
NHANES: National Health and Nutrition Examination Survey
OR: odds ratios
PA: physical activity
PAF: population attributable fraction
PHQ‐9: nine‐item Patient Health Care Questionnaire
RCS: restricted cubic spline
SNPs: single‐nucleotide polymorphisms
VATV: visceral adipose tissue volume

## 1 Introduction

Depression, characterized by persistent low mood, disinterest in daily activities, insomnia, and suicidal thoughts, is the most common mental health disorder (Qi et al. 2024). The World Health Organization reported that depression ranked as the third leading global health burden in 2018 and is expected to become the primary global health challenge by 2030 (Malhi and Mann 2018). Although therapeutic strategies have advanced, depression recurrence remains prevalent, affecting nearly 80% of patients with at least one subsequent episode. Given the rising global prevalence and adverse consequences, the prevention of depression has become an urgent issue.

Increasing research indicates that obesity substantially elevates the risk of depression (Jokela and Laakasuo 2023; Nong et al. 2024). A recent framework for managing obesity identified visceral adipose tissue as a key risk factor for health decline and emphasized the role of mental health in individuals with obesity (Busetto et al. 2024). Previous research confirmed a link between depression and higher levels of visceral adipose tissue (Cosan et al. 2021). Recent studies have identified several indicators of visceral adipose tissue that correlate with depressive symptoms, including the lipid accumulation product (Dai et al. 2024; Zhu et al. 2024), body roundness index (Zhang et al. 2024a, 2024b), and visceral adiposity index (Lei et al. 2022). While these indicators provide estimates of visceral adipose tissue volume (VATV), the definitive measurement is obtained through imaging techniques like dual‐energy x‐ray absorptiometry (DXA) and magnetic resonance imaging (Fang et al. 2018; Kaul et al. 2012).

Prior research consistently shows that individuals with increased VATV exhibit a higher prevalence of depression. However, these studies exhibited limitations in their study populations regarding age (Coryell et al. 2016; Milaneschi et al. 2012), gender (Pasco et al. 2023), race (Cameron et al. 2019), and income level (Cho et al. 2019), which may restrict their representativeness of the entire population. Additionally, numerous studies have shown that physical activity (PA) can significantly decrease the likelihood of depression (Noetel et al. 2024; Pearce et al. 2022). Research indicates that sedentary behavior is linked to a higher risk of depression (Yang et al. 2024; Zhou et al. 2024). Based on the factors mentioned above, a research gap exists: the insufficient exploration of the combined impact of VATV on health outcomes, particularly its effects on the depression process. This gap is especially significant due to the potential of outdoor PA engagement in reducing VATV.

Our study utilized large‐sample, representative data from the National Health and Nutrition Examination Survey (NHANES) database to assess the correlation between VATV and depression. Given that the studies investigating the connection between VATV and depression mentioned above are observational epidemiological studies, their ability to establish causal relationships is limited (Lawlor et al. 2008). Therefore, we introduced Mendelian randomization (MR) which effectively mitigates residual confounding and potential reverse causality, to further investigate the causal relationship (Birney 2022). Previous MR studies on obesity and depressive symptoms have focused on indicators such as body mass index or body fat mass (Speed et al. 2019; Tyrrell et al. 2019; Yan et al. 2024), and no MR study has reported the relationship between VATV and depression. Our study integrated an observational study with MR analysis to demonstrate the association between VATV and depression and further investigated the synergistic impact of VATV and PA on depression.

## 2 Materials and Methods

### 2.1 Study Population

This study used data from the NHANES, a cross‐sectional survey designed to assess the health and nutritional status of both adults and children in the United States. Since 1999, it has been conducted biennially and received approval from the National Center for Health Statistics Research Ethics Review Board. Informed consent was obtained from all participants (Zipf et al. 2013).

Participants were selected from four cycles of NHANES, from 2011 to 2018, aged between 20 and 59 years, and weighted to reflect the noninstitutionalized civilian population of the United States. Following the exclusion of participants with missing data on VATV, depression, and other covariates, the eligible participants were included in the study. Figure S1 illustrates the participant inclusion and exclusion procedures applied in this study.

### 2.2 Measurement of VATV and PA

VATV was measured at the approximate interstitial position of the L4 and L5 vertebrae by Hologic APEX software, which is used in DXA scan analysis. PA status was obtained from a questionnaire. Vigorous activities are defined as those causing substantial increases in breathing or heart rate, like running or playing basketball, and are maintained for at least 10 consecutive minutes. Moderate activities, such as brisk walking, cycling, swimming, or volleyball, are defined as those that slightly elevate breathing or heart rate and are performed continuously for at least 10 min. Regular PA was defined as the inclusion of either vigorous or moderate activities.

### 2.3 Assessment of Depression

The nine‐item Patient Health Questionnaire (PHQ‐9) scale was used to measure depression (Negeri et al. 2021; Spitzer et al. 1999). This questionnaire assesses the frequency of symptoms experienced during the preceding 2 weeks, using a scale from 0 (indicating “not at all”) to 3 (indicating “nearly every day”). The total scores can range from 0 to 27. In this study, clinically significant depressive symptoms were defined as a score of ≥ 10, demonstrating a sensitivity of 74% and a specificity of 91% (Song et al. 2024).

### 2.4 Covariates

The demographic variables included age, sex, race, poverty income ratio, and educational level. The questionnaire data included smoking status, alcohol status, and recreational physical activity level. Hypertension was characterized by a blood pressure of at least 130/85 mmHg or the use of antihypertensive medication. Diabetes status was assessed based on glycated hemoglobin levels (≥ 6.5%), fasting plasma glucose levels (≥ 126 mg/dL), or a confirmed diagnosis or treatment with antidiabetic medication. The collection and measurement of triglycerides and cholesterol were conducted at the NHANES Mobile Examination Center.

### 2.5 Genome‐Wide Association Studies (GWAS) Sources

The instrumental variables (IVs) in this study were derived from the MRC‐Integrative Epidemiology Unit (IEU), involving 32,860 European samples (IEU GWAS ID: ebi‐a‐GCST90016671). The single‐nucleotide polymorphisms (SNPs) were identified for MR analysis by selecting those strongly associated with VATV, using a significance threshold of *p* < 5 × 10−6. SNPs in linkage disequilibrium were excluded using parameters of *r* ^2^ < 0.001 and a distance of 10,000 kb. The *F* statistic was calculated for each SNP, with values exceeding 10 identified as robust instrumental variables, demonstrating a strong association with VATV (Papadimitriou et al. 2020). The *F* statistic is calculated as follows:

$$F=R^{2}\times \left(N-2\right)/1-R^{2}$$

In this equation, *N* represents the sample size, and *R* ^2^ denotes the variance in exposure accounted for by the chosen SNPs. The *R* ^2^ statistic is calculated as follows:

$$\begin{array}{ccc} \;R^{2} & & =2\times \mathrm{EAF}\times \left(1-\mathrm{EAF}\right)\times \beta^{2}/2\times \mathrm{EAF}\times \left(1-\mathrm{EAF}\right)\times \beta^{2}\\ & & +2\times \mathrm{EAF}\times \left(1-\mathrm{EAF}\right)\times N\times \mathrm{SE}\left(\beta^{2}\right) \end{array}$$

In this formula, *N* represents the sample size, EAF denotes the effect allele frequency, *β* indicates the SNPs' effect on exposure, and SE stands for the standard error.

The GWAS data for depression were obtained from MRC‐IEU, involving 180,866 European samples (IEU GWAS ID: ebi‐a‐GCST003769), which identified 6,019,632 independent genetic variants associated with depression. Figure S2 provides an overview of the research design.

### 2.6 Statistical Analysis

To address potential biases, data with missing covariates were excluded, and baseline demographic characteristics of both included and excluded groups were compared using standardized differences, considering differences below 10% as negligible (Table S1) (Gao et al. 2024; Muanda et al. 2019). The baseline characteristics of the participants included in the study were summarized using median values and interquartile range (IQR) (continuous variables; presented as the median [IQR]) or proportions (categorical variables; expressed as *N* [%]). Due to the complex probability cluster design used in NHANES, all statistical analyses in this study accounted for weights. Weighted multivariable‐adjusted logistic regression was used to evaluate the combined effects of VATV and PA on depression, with findings expressed as odds ratios (OR) and 95% confidence intervals (CI). Due to the positively skewed distribution, VATV was ln‐transformed. Covariates for multivariable analysis were selected based on theoretical insights from existing literature, aligning with recommendations rather than relying on statistical criteria (Hernán et al. 2002). Model 1 did not include confounder adjustments. Model 2 incorporated adjustments for demographic covariates such as age, sex, race, marital status, and family income. Model 3 extended these adjustments by also accounting for educational level, alcohol consumption, smoking status, diabetes, hypertension, triglycerides, and cholesterol levels. A multivariate‐adjusted restricted cubic spline (RCS) analysis was employed to investigate the dose‐response relationship. Subsequently, a threshold effect analysis was performed to determine potential cut‐off points. The population attributable fraction (PAF) was employed to calculate the proportion of depression that could be prevented if exposure (VATV and/or PA) were eliminated (Lin and Chen 2019; Liu et al. 2024). Subgroup analysis was conducted to assess potential moderating effects of age, sex, race, poverty income ratio, education level, smoking status, alcohol status, activity level, and comorbidities. Sensitivity analysis excluding the population using antidepressants was utilized to confirm the robustness of the results.

The TwoSampleMR package was utilized for all MR analyses (Emdin et al. 2017). We used the inverse‐variance weighted (IVW) method as the primary analysis. To check the robustness of the results, we employed MR Egger and weighted median methods for additional analysis. These methods entailed distinct validity assumptions for IVs (Zhao et al. 2023). The following sensitivity analyses were conducted: Cochran's Q test assessed heterogeneity in effect sizes across genetic instrumental variables, the MR‐PRESSO test identified and removed heterogeneous SNPs, the MR Egger regression intercept evaluated vertical pleiotropy, and leave‐one‐out analysis assessed the impact of individual SNPs on the MR estimate. All analyses employed R software (version 4.2.0), with statistical significance set at *p* < 0.05.

## 3 Results

### 3.1 Baseline Characteristics of NHANES

Among 7460 eligible adults with complete data on visceral fat and depression, the median (IQR) age was 40 (29, 49) years, 3495 (47%) participants were female; 1022 (9%) of participants were Mexican American, a total of 1584 participants (10%) were non‐Hispanic Black, while 2932 participants (66%) were non‐Hispanic White; 2750 (35%) had hypertension and 728 (8%) had diabetes. Among the included participants, 623 (8%) were defined as having depression. Table 1 presents a comparison of the baseline characteristics between individuals with and without depression. Participants with depression were more likely to be female, less likely to be married, had lower income, had lower educational levels, and were more inclined to smoke and drink alcohol. They also had a higher likelihood of having hypertension or diabetes, engaged in fewer physical activities, and exhibited elevated triglyceride and lnVATV levels. The characteristics of the study population, categorized by the quartiles of VATV, are presented in Table S2.

**TABLE 1:** Characteristics of the included participants from NHANES 2011–2018.

	Overall	Depression		
**Characteristic**	*N* = 7,460^a^	**Non‐depression**, *N* = 6,837	**Depression**, *N* = 623	***p* Value** ^b^
Age (years)	40 (29, 49)	40 (29, 49)	40 (30, 50)	0.7
Sex (%)				**<0.001**
Female	3495 (47%)	3107 (46%)	388 (62%)	
Male	3965 (53%)	3730 (54%)	235 (38%)	
Race/ethnicity (%)				**0.003**
Mexican American	1022 (10%)	966 (10%)	56 (6%)	
Non‐Hispanic White	2932 (66%)	2655 (66%)	277 (64%)	
Non‐Hispanic Black	1584 (10%)	1455 (10%)	129 (11%)	
Other/multiracial	1922 (14%)	1761 (14%)	161 (19%)	
Marital status (%)				**<0.001**
Married	3487 (51%)	3306 (53%)	181 (34%)	
Widowed	103 (1%)	79 (1%)	24 (4%)	
Divorced	718 (10%)	610 (9%)	108 (15%)	
Separated	254 (3%)	205 (2%)	49 (6%)	
Never married	2010 (24%)	1831 (24%)	179 (27%)	
Living with partner	888 (11%)	806 (11%)	82 (14%)	
PIR	3.22 (1.57, 5.00)	3.32 (1.69, 5.00)	1.71 (0.92, 3.74)	**<0.001**
Education level (%)				**<0.001**
<High school	1089 (10%)	952 (10%)	137 (17%)	
≥High school	6371 (90%)	5885 (90%)	486 (83%)	
Smoking status (%)				**<0.001**
Never smoker	4199 (56%)	3989 (58%)	210 (34%)	
Former smoker	1379 (21%)	1265 (21%)	114 (18%)	
Current smoker	1882 (23%)	1583 (21%)	299 (48%)	
Alcohol status (%)				**<0.001**
Non‐excessive alcohol	3224 (43%)	3009 (44%)	214 (33%)	
Excessive alcohol	4237 (57%)	3828 (56%)	409 (67%)	
Diabetes (%)				**0.020**
Non‐diabetes	6732 (92%)	6192 (92%)	540 (89%)	
Diabetes	728 (8%)	645 (8%)	83 (11%)	
Hypertension (%)				**0.003**
Non‐hypertension	4710 (65%)	4368 (65%)	342 (57%)	
Hypertension	2750 (35%)	2469 (35%)	281 (43%)	
Physical activity (%)				**<0.001**
Non‐regular activities	3124 (38%)	2735 (36%)	389 (59%)	
Regular activities	4336 (62%)	4102 (64%)	234 (41%)	
Triglycerides (mg/dL)	115 (76, 180)	114 (76, 178)	133 (85, 198)	**<0.001**
Cholesterol (mg/dL)	190 (165, 218)	190 (165, 218)	194 (166, 223)	0.080
lnVATV (cm^3^)	6.20 (5.71, 6.58)	6.19 (5.70, 6.58)	6.28 (5.80, 6.69)	**0.002**

Abbreviations: PIR, Ratio of family income to poverty; VATV, visceral adipose tissue volume.

^a^ Median (IQR) for continuous; *n* (%) for categorical.

^b^ Independent sample *t*‐test for continuous variables and the *χ* ^2^ test for categorical variables.

### 3.2 Association of VATV and PA Status With Depression

**FIGURE 1:**
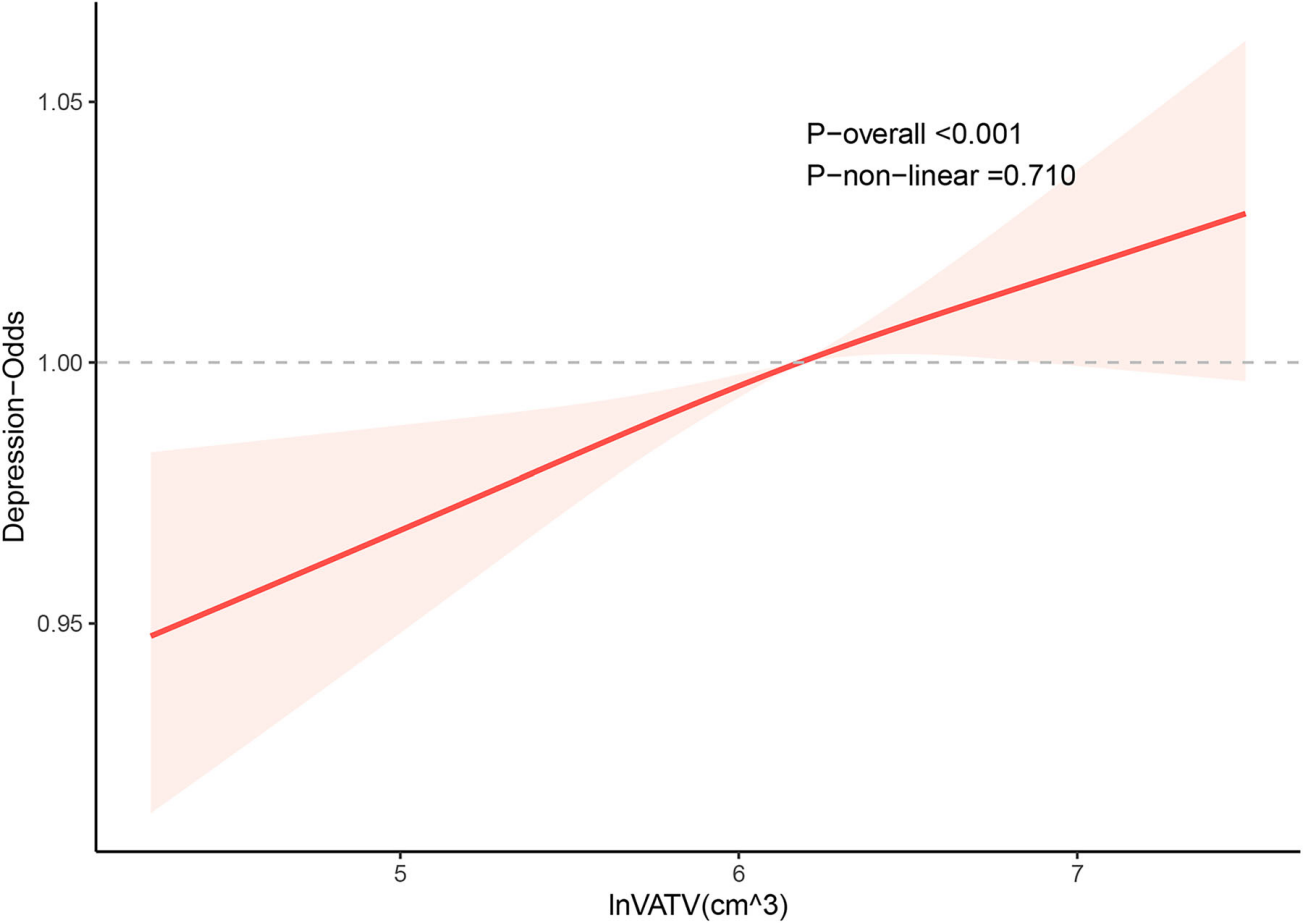
Restricted cubic spline plots of the association between lnVATV and depression. Adjusted for age, gender, race, marital status, family income, educational level, alcohol status, smoking status, diabetes, hypertension, triglycerides, and cholesterol level. CI, confidence interval; OR, odds ratio; VATV, visceral adipose tissue volume.

Weighted multivariable logistic regression models were employed to explore the relationship between VATV and depression. Table 2 shows that in Model 3, after adjusting for various variables, the OR for depression in the highest quartile of lnVATV was 1.04 (95% CI, 1.01–1.07) compared to the reference group, with a significant trend (*P* for trend < 0.05). Adequate PA was associated with a reduced risk of depression (OR, 0.52; 95% CI, 0.42–0.65). These results were robust across all models. Figure 1 illustrates the relationship between lnVATV and depression in terms of dose‐response. After adjusting for multiple variables, RCS revealed no significant nonlinear associations between lnVATV and depression (*P* for nonlinear = 0.710, *P* for overall < 0.001).

**TABLE 2:** Association of lnVATV levels and PA status with depression.

	Model 1^b^ OR (95% CI)	*p* Value	Model 2^c^ OR (95% CI)	*p* Value	Model 3^d^ OR (95% CI)	*p* Value
lnVATV (continuous)	1.30 (1.11, 1.54)	**0.002**	1.54 (1.27, 1.87)	**<0.001**	1.40 (1.15, 1.71)	**0.001**
Quartile of lnVATV						
Q1	Ref.^a^		Ref.		Ref.	
Q2	1.01 (0.99, 1.04)	0.303	1.03 (1.00, 1.05)	**0.036**	1.02 (1.00, 1.05)	**0.037**
Q3	1.01 (0.99, 1.03)	0.239	1.03 (1.01, 1.04)	**0.002**	1.02 (1.01, 1.04)	**0.009**
Q4	1.03 (1.01, 1.06)	**0.006**	1.05 (1.02, 1.08)	**<0.001**	1.04 (1.01, 1.07)	**0.004**
P for trend	**0.005**		**<0.001**		**0.011**	
PA	0.39 (0.31, 0.48)	**<0.001**	0.46 (0.37, 0.58)	**<0.001**	0.52 (0.42, 0.65)	**<0.001**

Abbreviations: VATV, visceral adipose tissue volume; PA, physical activity; OR, odds ratio; CI, confidence interval.

^a^ Ref: reference.

^b^ Model 1 was non‐adjusted model.

^c^ Model 2 was adjusted for age, gender, race, marital status, and family income.

^d^ Model 3 was further adjusted for educational level, alcoholic status, smoking status, diabetes, hypertension, triglycerides, and cholesterol level.

**FIGURE 2:**
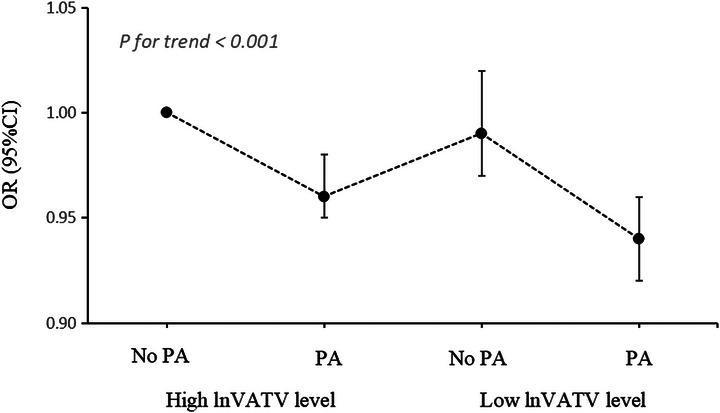
Joint association of lnVATV levels and PA status with depression. CI, confidence interval; OR, odds ratio; PA, physical activity; VATV, visceral adipose tissue volume.

Joint analyses indicated that participants exhibiting elevated lnVATV levels combined with insufficient physical activity faced the greatest risk of depression in the fully adjusted model. When compared to the group characterized by high lnVATV levels and insufficient PA, the odds ratio (OR) for depression in individuals with high lnVATV and adequate PA was 0.96 (95% CI: 0.95–0.98). Additionally, in comparison to other groups, individuals with low lnVATV and adequate PA exhibited the lowest depression risks (OR, 0.94; 95% CI, 0.92–0.96) (Table S3 and Figure 2). Furthermore, PAF analysis was conducted to estimate the proportion of participants **at** risk of depression that could be **prevented** if high VATV and/or PA deficiency were eliminated. As shown in Table 3, 8.8% and 27.6% of the decrease in depression were linked to low lnVATV levels and adequate PA, respectively. Furthermore, 30.2% of the reduction in depression was explained by the combination of low lnVATV levels and regular engagement in adequate PA.

**TABLE 3:** PAF of lnVATV levels and PA status with depression.

	Model 1^a^ PAF (95% CI)	*p* Value	Model 2^b^ PAF (95% CI)	*p* Value	Model 3^c^ PAF (95% CI)	*p* Value
lnVATV	−0.110 (−0.169, −0.051)	**<0.001**	−0.129 (−0.197, −0.061)	**<0.001**	−0.088 (−0.154, −0.021)	**0.009**
PA	−0.491 (−0.579, −0.403)	**<0.001**	−0.341 (−0.427, −0.255)	**<0.001**	−0.276 (−0.359, −0.194)	**<0.001**
lnVATV and PA	−0.504 (−0.631, −0.377)	**<0.001**	−0.389 (−0.520, −0.259)	**<0.001**	−0.302 (−0.426, −0.178)	**<0.001**

Abbreviations: CI, confidence interval; PA, physical activity; PAF, population attributable fraction; VATV, visceral adipose tissue volume.

^a^ Model 1 was non‐adjusted model.

^b^ Model 2 was adjusted for age, gender, race, marital status, and family income.

^c^ Model 3 was further adjusted for educational level, alcoholic status, smoking status, diabetes, hypertension, triglycerides and cholesterol level.

### 3.3 Subgroup Analysis and Sensitivity Analysis

**FIGURE 3:**
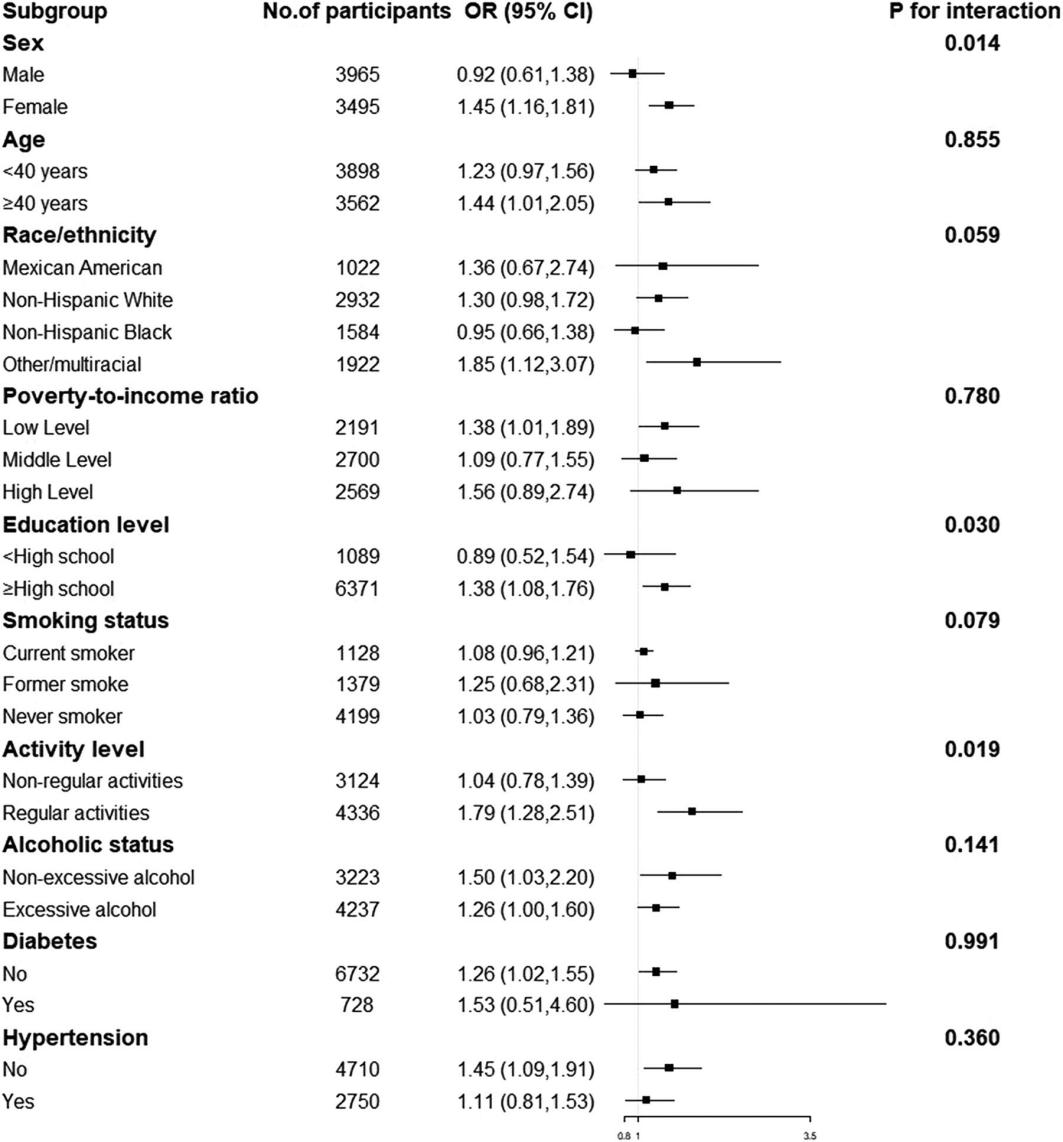
Forest plots for subgroup analysis. Subgroup analysis was stratified by age, sex, race, poverty income ratio, education level, smoking status, activity level, alcohol status, and comorbidities. CI, confidence interval; OR, odds ratio.

Subgroup analysis by age, sex, race, poverty income ratio, education level, smoking status, alcohol status, activity level, and comorbidities confirmed consistent results with the main findings (Figure 3). A significant interaction between lnVATV and PA was observed concerning depression risk (*P* for interaction < 0.05). In addition, sex and education had an interaction with lnVATV in relation to depression, suggesting that these relationships were stronger in females and among individuals with higher levels of education.

The participants were categorized into two distinct groups for each sex, and weighted multivariable‐adjusted logistic regression analyses were subsequently conducted. Table S4 shows that lnVATV is associated with a higher risk of depression in female participants (OR, 1.54; 95% CI, 1.24–1.91, *p* < 0.001) after adjusting for age, race, marital status, family income, educational level, alcohol status, smoking status, diabetes, hypertension, triglycerides, and cholesterol level. However, the similar relationship was not significant in males (OR, 0.98; 95% CI, 0.65–1.48, *p* = 0.940).

Table S5 presents the outcome of the sensitivity analysis. After excluding participants treated with antidepressant medication (*n* = 43), lnVATV is associated with depression (OR, 1.42; 95% CI, 1.17–1.73, *p* < 0.001). The result is robust in all models.

### 3.4 MR Analysis of VATV and Depression

**FIGURE 4:**
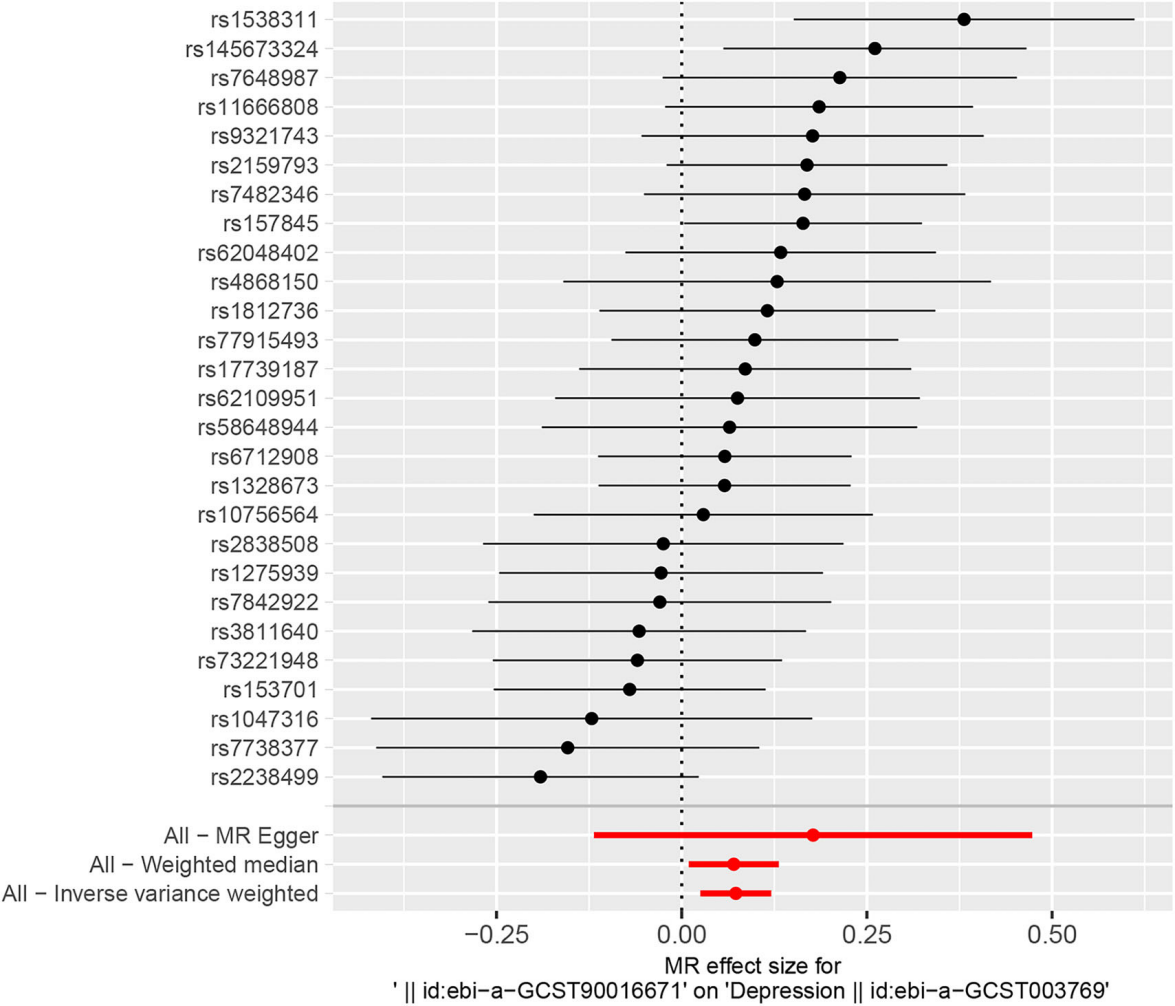
Mendelian randomization effect size for VATV on depression. VATV, visceral adipose tissue volume.

Detailed summary information of the SNPs was given in Table S6. Figure 4 and Table S7 demonstrate a significant causal link between VATV and depression risk using IVW and weighted median methods, while the MR Egger method did not show significance. The pooled OR for depression risk per unit change in genetic prediction was 1.08 (95%CI, 1.03–1.13) using IVW, and 1.07 (95%CI, 1.01–1.14) with the weighted median. The reliability of our results was validated by sensitivity analyses. This study found no evidence of heterogeneity (P Q > 0.05) or pleiotropy (*P* intercept > 0.05) as indicated in Table S8. The robustness of these results was further confirmed by leave‐one‐out analysis (Figure S3). The scatter plot and funnel plot are displayed in Figures S4 and S5.

## 4 Discussion

Our findings indicated that high VATV levels were significantly associated with an increase in depression prevalence. The intricate relationship between visceral adipose tissue and depression is shaped by factors including age, race, family income, PA, educational level, and smoking. Our findings indicate that integrating sufficient PA with low VATV is more effective in decreasing depression prevalence than merely lowering VATV. More importantly, MR analysis confirmed the causal effect of VATV and depression. These results highlight the intricate relationship between VATV and depression, offering insights that may guide future investigations, potentially contributing to the development of clinical guidelines. To the best of our knowledge, this study represents the first attempt to explore the joint association of VATV and PA status on depression.

Obesity and depression are two major health conditions that have a profound impact on both individuals and society at large (Milaneschi et al. 2019). There is a consensus that visceral fat presents a higher health risk compared to subcutaneous fat, increasing disease susceptibility. Our findings showed a positive association between VATV and depression, which was in substantial agreement with previous studies (Fei et al. 2024; Lei et al. 2022; Shen et al. 2024; Zhang et al. 2024b). Potential mechanisms of association between visceral obesity and depression may be multifactorial. First, visceral obesity can hyperactivate the hypothalamic‐pituitary‐adrenal (HPA) axis through various pathways, which is widely recognized to be associated with depression (André et al. 2014; Dwyer et al. 2020; Shen et al. 2021). Second, the inflammatory response has been identified as a potential mechanism connecting visceral obesity and depression (Beurel et al. 2020; Franklin et al. 2018; Torres et al. 2024). Furthermore, gut microbiota significantly influences this relationship (Schachter et al. 2018; Shantaram et al. 2024).

The current study focused on VATV as an independent variable rather than being limited to an index calculated indirectly, and DXA was used to quantify visceral fat more directly and accurately. Previous studies have explored the link between depression and visceral adipose tissue assessed through imaging techniques. A Korean study identified a link between depression and visceral adipose tissue in women, although it did not include low‐income populations in its sample (Cho et al. 2019). However, our study found that the link was even stronger among low‐income participants. Similarly, Milaneschi et al. (2012) focused solely on older adults aged 70–79 years, while younger people were more vulnerable to depression (Varma et al. 2021). In addition, previous studies on depression and visceral adipose tissue measured by DXA have notable limitations. For example, Pasco et al. (2023) included only women, Coryell et al. (2016) restricted their sample to individuals aged 15–20 years, and Cameron et al. (2019) lacked ethnic diversity. In this study, a broader population was included, further verifying and extending the conclusions of previous studies, that high VATV is associated with an increased prevalence of depression.

Our study indicated that the combination of effective PA with VATV was significantly linked to a greater reduction in depression prevalence compared to low VATV levels alone. It is noteworthy that regular PA has been shown to have beneficial effects on depression development. A recent network meta‐analysis including 218 studies showed that exercise was an effective treatment for depression (Noetel et al. 2024). Additionally, an MR study confirmed that increased PA was an effective strategy for preventing depression (Choi et al. 2019). PA may affect depression through various psychosocial and biological mechanisms, including the production of brain‐derived neurotrophic factors, stimulation of neuroplasticity in specific brain regions, reduction of inflammation and oxidative stress, and enhancement of self‐esteem (Kandola et al. 2019; Kunugi 2023; Ross et al. 2023; Zarza‐Rebollo et al. 2024). In addition, the interaction analysis also indicated a significant interaction effect between VATV and sex on depression, suggesting that VATV has a greater influence on depression in females. This may be attributed to sex differences in hormone levels; fluctuations in estrogen are thought to increase the risk of depression in females (Barth et al. 2023; Steiner and Berry 2022).

Our study had several strengths. Utilizing a complex multi‐stage probability sampling method, we extracted a substantial sample from the NHANES database, ensuring accurate representation of the non‐institutionalized population and enhancing the external generalizability of our findings. Second, we identified a combined impact of VATV and PA on depression, highlighting the importance of addressing both obesity and physical activity in managing depression. More significantly, we integrated both observational data and MR analysis. While causal inference based solely on the NHANES study was not feasible, the application of MR analysis helps overcome the inherent limitations associated with observational studies. However, there were limitations to consider. First, observational studies may be subject to self‐reported recall bias. Second, the study might have overlooked certain confounding variables, including comorbidities, which were not accounted for in the analysis. These factors could have significant implications for the interpretation of the results. In addition, since the information about antidepressant medication was not available in the MR analysis, its potential impact could not be accounted for. Finally, we would like to point out that our data were limited to the US and European populations, and the observational study population was limited to 20–59 years old due to the constraints of those who underwent DXA examinations. Consequently, our results may be biased and not applicable to other demographic groups. Due to the limitations inherent in cross‐sectional studies, additional validation through evidence‐based methodologies, such as randomized controlled trials and longitudinal research, is necessary. Furthermore, considering the gender‐specific effects of lnVATV, further research is needed to examine estrogen's potential role in the gender‐specific effects of lnVATV.

## 5 Conclusion

In summary, the current findings highlight a positive association between VATV and depression. In addition, individuals who exhibited sufficient PA and lower levels of VATV were more closely associated with a reduced incidence of depression. This study highlights the importance of both VATV and PA in the management of depression, providing valuable contributions to the current understanding in this area. Further research involving clinical populations is necessary to validate these results.

## Author Contributions

G.L.X. and F.H.Y. contributed to conception, design, and the acquisition and analysis of data. L.Y.H. and L.W. contributed to the interpretation of data. G.L.X. and L.W. drafted the manuscript. X.L.Y. revised the manuscript and supervised the study. All authors approved the submitted version of the manuscript.

## Funding

The authors have nothing to report.

## Ethics Statement

The protocols of NHANES were approved by the institutional review board of the National Center for Health Statistics. NHANES has obtained written informed consent from all participants.

## Conflicts of Interest

The authors declare no conflicts of interest.

## Supporting information




**Supplementary Material**: brb371392‐sup‐0001‐SuppMat.docx

## Data Availability

The datasets generated and analyzed in the current study are available at the NHANES website: https://www.cdc.gov/nchs/nhanes/index.htm, and the IEU OpenGWAS database: https://gwas.mrcieu.ac.uk/. The other datasets generated and/or analyzed during the current study are publicly available and included in this published article and its supplementary information files.
