# Supplementary material for: Association Between Visceral Adipose Tissue and Depression Risk With Both Observational and Genetic Evidence
Source: Brain Behav. 2026 Apr 23;16(4):e71392. doi: 10.1002/brb3.71392 (PMC13103535; doi:10.1002/brb3.71392)
Supplement: Supplementary file 1 — Supplementary Material: brb371392‐sup‐0001‐SuppMat.docx [file BRB3-16-e71392-s001.docx]

**Supplementary Table 1**

Baseline characteristics of excluded and included data.

| **Characteristic** | **Excluded Data**, N = 3,707^a^ | **Included Data**, N = 7,460 | **Standardized Difference (%)**^b^ |
| --- | --- | --- | --- |
| Age (%) |  |  |  |
| <40 years | 1,646 (46%) | 3,898 (50%) | 8 |
| ≥40 years | 2,061 (54%) | 3,562 (50%) | 8 |
| Sex (%) |  |  |  |
| Female | 1,948 (51%) | 3,495 (47%) | 8 |
| Male | 1,759 (49%) | 3,965 (53%) | 8 |
| Race/Ethnicity (%) |  |  |  |
| Mexican American | 566 (11%) | 1,022 (10%) | 3 |
| Other/multiracial | 3,141 (89%) | 6,438 (90%) | 3 |
| Marital status (%) |  |  |  |
| Married | 1,885 (52%) | 3,487 (51%) | 2 |
| Widowed | 44 (1%) | 103 (1%) | 0 |
| Divorced | 312 (9%) | 718 (10%) | 3 |
| Separated | 144 (3%) | 254 (3%) | 0 |
| Never married | 953 (25%) | 2,010 (24%) | 2 |
| Living with partner | 368 (10%) | 888 (11%) | 3 |
| Diabetes (%) |  |  |  |
| Non-diabetes | 3,239 (90%) | 6,732 (92%) | 7 |
| Diabetes | 468 (10%) | 728 (8%) | 7 |
| Hypertension (%) |  |  |  |
| Non-hypertension | 2,253 (62%) | 4,710 (65%) | 6 |
| Hypertension | 1,454 (38%) | 2,750 (35%) | 6 |
| ^a^ median (IQR) for continuous; n (%) for categorical | | | |
| ^b^ The difference between the groups divided by the pooled standard deviation; a value lower than 10% is interpreted as a meaningless difference. | | | |

**Supplementary Table 2**

Characteristics of study population according to quartiles of visceral adipose tissue volume.

| **Characteristic** | **Visceral adipose tissue volume** | | | |
| --- | --- | --- | --- | --- |
|  | **Quartile 1**, N = 1,893^a^ | **Quartile 2**, N = 1,928^a^ | **Quartile 3**, N = 1,914^a^ | **Quartile 4**, N = 1,725^a^ |
| Age (years) | 28 (24, 39) | 36 (28, 46) | 43 (34, 51) | 47 (39, 54) |
| Sex (%) |  |  |  |  |
| Female | 1,070 (60%) | 876 (45%) | 833 (44%) | 716 (39%) |
| Male | 823 (40%) | 1,052 (55%) | 1,081 (56%) | 1,009 (61%) |
| Race/Ethnicity (%) |  |  |  |  |
| Mexican American | 114 (4.7%) | 228 (8.8%) | 312 (11%) | 368 (12%) |
| Non-Hispanic White | 747 (66%) | 687 (63%) | 707 (63%) | 791 (71%) |
| Non-Hispanic Black | 511 (14%) | 466 (12%) | 389 (10%) | 218 (5.4%) |
| Other/multiracial | 521 (15%) | 547 (16%) | 506 (15%) | 348 (11%) |
| PIR | 2.97 (1.45, 5.00) | 3.32 (1.61, 5.00) | 3.21 (1.62, 5.00) | 3.30 (1.61, 5.00) |
| Education Level (%) |  |  |  |  |
| <High school | 184 (7%) | 276 (10%) | 323 (11%) | 306 (12%) |
| ≥High school | 1,709 (93%) | 1,652 (90%) | 1,591 (89%) | 1,419 (88%) |
| Marital |  |  |  |  |
| Married | 604 (36%) | 894 (51%) | 1,022 (58%) | 967 (59%) |
| Widowed | 11 (1%) | 25 (1%) | 27 (1%) | 40 (2%) |
| Divorced | 115 (6%) | 167 (8%) | 213 (12%) | 223 (13%) |
| Separated | 44 (1%) | 65 (3%) | 77 (3%) | 68 (3%) |
| Never married | 860 (42%) | 542 (26%) | 350 (16%) | 258 (14%) |
| Living with partner | 259 (14%) | 235 (12%) | 225 (10%) | 169 (9%) |
| Smoking status (%) |  |  |  |  |
| Never smoker | 1,184 (63%) | 1,115 (58%) | 1,072 (56%) | 828 (48%) |
| Former smoker | 237 (15%) | 332 (19%) | 374 (22%) | 436 (29%) |
| Current smoker | 472 (23%) | 481 (23%) | 468 (22%) | 461 (23%) |
| Alcohol status (%) |  |  |  |  |
| Non-excessive alcohol | 797 (39%) | 870 (45%) | 838 (45%) | 718 (43%) |
| Excessive alcohol | 1,096 (61%) | 1,058 (55%) | 1,076 (55%) | 1,007 (57%) |
| Diabetes (%) |  |  |  |  |
| Diabetes | 25 (1%) | 94 (3%) | 205 (8%) | 404 (19%) |
| Non-diabetes | 1,868 (99%) | 1,834 (97%) | 1,709 (92%) | 1,321 (81%) |
| Hypertension (%) |  |  |  |  |
| Hypertension | 331 (17%) | 544 (23%) | 864 (43%) | 1,011 (59%) |
| Non-hypertension | 1,562 (83%) | 1,384 (77%) | 1,050 (57%) | 714 (41%) |
| Physical activity (%) |  |  |  |  |
| Non-Regular activities | 570 (25%) | 730 (33%) | 893 (42%) | 931 (52%) |
| Regular activities | 1,323 (75%) | 1,198 (67%) | 1,021 (58%) | 794 (48%) |
| Triglycerides (mg/dL) | 78 (58, 108) | 102 (71, 154) | 133 (90, 203) | 173 (118, 257) |
| Cholesterol (mg/dL) | 173 (154, 196) | 188 (165, 213) | 197 (173, 228) | 203 (176, 231) |
| Depression |  |  |  |  |
| Depression | 127 (6%) | 155 (8%) | 148 (7%) | 193 (10%) |
| Non-depression | 1,766 (94%) | 1,773 (92%) | 1,766 (93%) | 1,532 (90%) |
| ^a^ Median (IQR); n (unweighted) (%);  PIR, Ratio of family income to poverty. | | | | |

**Supplementary Table 3**

Joint association of lnVATV levels and PA status with depression.

|  | | **Model 1**^b^ **OR (95% CI)** | **P Value** | **Model 2**^c^ **OR (95% CI)** | **P Value** | **Model 3**^d^ **OR (95% CI)** | **P Value** |
| --- | --- | --- | --- | --- | --- | --- | --- |
| lnVATV high level | No PA | Ref.^a^ |  | Ref.^a^ |  | Ref.^a^ |  |
|  | PA | 0.94 (0.92, 0.96) | **<0.001** | 0.96 (0.94, 0.97) | **<0.001** | 0.96 (0.95, 0.98) | **<0.001** |
| lnVATV low level | No PA | 1.01 (0.98, 1.04) | 0.428 | 1.00 (0.97, 1.02) | 0.713 | 0.99 (0.97, 1.02) | 0.678 |
|  | PA | 0.93 (0.91, 0.94) | **<0.001** | 0.93 (0.91, 0.95) | **<0.001** | 0.94 (0.92, 0.96) | **<0.001** |
|  | P for trend | **<0.001** |  | **<0.001** |  | **<0.001** |  |
| ^a^ Ref: reference.  ^b^ Model 1 was non-adjusted model.  ^c^ Model 2 was adjusted for age, gender, race, marital status, and family income.  ^d^ Model 3 adjusted further adjusted for educational level, alcoholic status, smoking status, diabetes, hypertension, triglycerides and cholesterol level.  VATV, visceral adipose tissue volume; PA, physical activity; OR, odds ratio; CI, confidence interval. | | | | | | | |

**Supplementary Table 4**

The different relationship between lnVATV and depression in sex subgroup.

|  | **Male OR (95% CI) ^b^** | **P Value** | **Female OR (95% CI)** | **P Value** |
| --- | --- | --- | --- | --- |
| lnVATV (continuous) | 0.98 (0.65, 1.48) | 0.940 | 1.54 (1.24, 1.91) | **<0.001** |
| Quartile of CDAI |  |  |  |  |
| Q1 | Ref.^a^ | | Ref. | |
| Q2 | 1.00 (0.98, 1.03) | 0.754 | 1.03 (0.99, 1.06) | 0.112 |
| Q3 | 1.01 (0.98, 1.03) | 0.471 | 1.04 (1.01, 1.07) | **0.013** |
| Q4 | 1.00 (0.97, 1.03) | 0.977 | 1.07 (1.04, 1.11) | **<0.001** |
| P for trend | 0.909 |  | **<0.001** |  |
| ^a^ Ref: reference;  ^b^ Model was adjusted for age, gender, race, marital status, family income, educational level, alcoholic status, smoking status, diabetes, hypertension, triglycerides and cholesterol level;  VATV, visceral adipose tissue volume; OR, odds ratio; CI, confidence interval. | | | | |

**Supplementary Table 5**

Sensitivity analysis.

|  | **Model 1**^b^ **OR (95% CI)** | **P Value** | **Model 2**^c^ **OR (95% CI)** | **P Value** | **Model 3**^d^ **OR (95% CI)** | **P Value** |
| --- | --- | --- | --- | --- | --- | --- |
| lnVATV (continuous) | 1.32 (1.12, 1.56) | **0.002** | 1.55 (1.28, 1.88) | **<0.001** | 1.42 (1.17, 1.73) | **<0.001** |
| Quartile of lnVATV |  |  |  |  |  |  |
| Q1 | Ref.^a^ | | Ref. | | Ref. | |
| Q2 | 1.01 (0.99, 1.04) | 0.265 | 1.03 (1.00, 1.05) | **0.026** | 1.03 (1.00, 1.05) | **0.028** |
| Q3 | 1.01 (0.99, 1.03) | 0.209 | 1.03 (1.01, 1.04) | **0.002** | 1.02 (1.01, 1.04) | **0.006** |
| Q4 | 1.04 (1.01, 1.06) | **0.005** | 1.05 (1.02, 1.08) | **<0.001** | 1.04 (1.02, 1.07) | **0.004** |
| P for trend | **0.004** |  | **<0.001** |  | **0.010** |  |
| ^a^ Ref: reference.  ^b^ Model 1 was non-adjusted model.  ^c^ Model 2 was adjusted for age, gender, race, marital status, and family income.  ^d^ Model 3 adjusted further adjusted for educational level, alcoholic status, smoking status, diabetes, hypertension, triglycerides and cholesterol level.  VATV, visceral adipose tissue volume; OR, odds ratio; CI, confidence interval. | | | | | | |

**Supplementary Table 6**

The total VATV-related genetic variants used for the MR analysis.

| **Exposure** | **SNPs** | **Chr** | **Position** | **effect_allele** | **other_allele** | **EAF** | **Beta** | **SE** | **P value** | **R^2^** | **F-statistic** | **Explained variance (R^2^)** |
| --- | --- | --- | --- | --- | --- | --- | --- | --- | --- | --- | --- | --- |
| VATV | rs1047316 | 6 | 19837966 | C | T | 0.3105 | 0.0329 | 0.0072 | 4.20E-06 | 0.0091 | 302.9430 | 41.90%  41.90% |
| VATV | rs10756564 | 9 | 14514783 | T | C | 0.6213 | -0.0342 | 0.0068 | 4.70E-07 | 0.0099 | 327.9681 |  |
| VATV | rs11666808 | 19 | 18383506 | C | T | 0.6278 | -0.0377 | 0.0068 | 3.50E-08 | 0.0120 | 398.0589 |  |
| VATV | rs1275939 | 2 | 26957737 | A | G | 0.4710 | 0.0359 | 0.0066 | 5.10E-08 | 0.0108 | 359.9483 |  |
| VATV | rs1328673 | 13 | 47615594 | T | C | 0.3440 | 0.0346 | 0.0069 | 6.40E-07 | 0.0101 | 334.1755 |  |
| VATV | rs145673324 | 11 | 42818552 | G | T | 0.0788 | -0.0575 | 0.0122 | 2.60E-06 | 0.0274 | 924.5551 |  |
| VATV | rs153701 | 19 | 34003255 | C | T | 0.5476 | -0.0427 | 0.0067 | 1.40E-10 | 0.0153 | 510.2841 |  |
| VATV | rs1538311 | 10 | 76415509 | T | G | 0.4224 | -0.0341 | 0.0067 | 4.10E-07 | 0.0098 | 325.1054 |  |
| VATV | rs157845 | 5 | 55796639 | C | T | 0.7447 | -0.0488 | 0.0075 | 9.50E-11 | 0.0199 | 666.7927 |  |
| VATV | rs17739187 | 18 | 42749456 | A | C | 0.2470 | -0.0350 | 0.0076 | 4.60E-06 | 0.0103 | 342.2387 |  |
| VATV | rs1812736 | 8 | 76299138 | A | G | 0.8290 | 0.0432 | 0.0088 | 8.40E-07 | 0.0157 | 522.4290 |  |
| VATV | rs2159793 | 9 | 126701763 | C | T | 0.2187 | 0.0414 | 0.0080 | 2.40E-07 | 0.0144 | 478.7217 |  |
| VATV | rs2238499 | 16 | 24282599 | A | G | 0.2818 | 0.0367 | 0.0074 | 6.50E-07 | 0.0113 | 376.9991 |  |
| VATV | rs2838508 | 21 | 45586002 | T | G | 0.8086 | 0.0403 | 0.0084 | 1.50E-06 | 0.0136 | 453.6641 |  |
| VATV | rs3811640 | 2 | 112776979 | A | C | 0.2784 | -0.0348 | 0.0073 | 2.00E-06 | 0.0102 | 338.6853 |  |
| VATV | rs4868150 | 5 | 171624607 | A | G | 0.0875 | 0.0543 | 0.0116 | 3.00E-06 | 0.0245 | 825.0180 |  |
| VATV | rs58648944 | 15 | 67965102 | G | A | 0.5958 | 0.0310 | 0.0068 | 4.70E-06 | 0.0081 | 267.8641 |  |
| VATV | rs62048402 | 16 | 53803223 | A | G | 0.3995 | 0.0374 | 0.0067 | 3.00E-08 | 0.0118 | 391.2772 |  |
| VATV | rs62109951 | 19 | 29854071 | T | C | 0.2122 | -0.0398 | 0.0080 | 6.70E-07 | 0.0133 | 443.1481 |  |
| VATV | rs6712908 | 2 | 44357936 | T | C | 0.3727 | -0.0344 | 0.0068 | 4.30E-07 | 0.0100 | 330.4068 |  |
| VATV | rs73221948 | 8 | 25464670 | T | G | 0.2933 | -0.0502 | 0.0075 | 2.50E-11 | 0.0210 | 704.6618 |  |
| VATV | rs7482346 | 11 | 14192586 | C | T | 0.1080 | 0.0542 | 0.0106 | 2.90E-07 | 0.0244 | 822.2046 |  |
| VATV | rs7648987 | 3 | 49868455 | G | A | 0.4974 | 0.0328 | 0.0066 | 6.70E-07 | 0.0091 | 300.6668 |  |
| VATV | rs7738377 | 6 | 34595892 | A | G | 0.1339 | 0.0455 | 0.0097 | 2.60E-06 | 0.0173 | 578.4416 |  |
| VATV | rs77915493 | 5 | 132568984 | C | A | 0.1223 | 0.0506 | 0.0100 | 4.60E-07 | 0.0213 | 715.9865 |  |
| VATV | rs7842922 | 8 | 112344159 | G | T | 0.6875 | 0.0339 | 0.0071 | 1.80E-06 | 0.0097 | 321.1040 |  |
| VATV | rs9321743 | 6 | 139963957 | G | T | 0.3402 | 0.0340 | 0.0070 | 1.10E-06 | 0.0097 | 322.3635 |  |
| VATV, visceral adipose tissue volume; SNPs, single-nucleotide polymorphisms; Chr, chromosome; EAF, effect allele frequency; SE, standard error. | | | | | | | | | | | | |

**Supplementary Table 7**

OR estimates and 95% CI for Inverse variance weighted, MR Egger and weighted median.

| **Exposure** | **Outcome** | **Method** | **nSNPs** | **OR (95% CI)** | **P Value** |
| --- | --- | --- | --- | --- | --- |
| VATV | Depression | Inverse variance weighted | 27 | 1.08 (1.03, 1.13) | **0.003** |
|  |  | MR Egger | 27 | 1.94 (0.89, 1.61) | 0.251 |
|  |  | Weighted median | 27 | 1.07 (1.01, 1.14) | **0.021** |
| OR, odds ratio; CI, confidence interval; MR, mendelian randomization; SNPs, single nucleotide polymorphisms; VATV, visceral adipose tissue volume. | | | | | |

**Supplementary Table 8**

Sensitivity analysis for MR analysis.

| **Exposure** | **Outcome** | **Cochran Q statistic** | **Heterogeneity P value** | **MR-Egger**  **Intercept** | **Intercept**  **P value** | **MR-PRESSO Global test P value** |
| --- | --- | --- | --- | --- | --- | --- |
| VATV | Depression | 35.240 | 0.107 | -0.004 | 0.490 | 0.119 |
| MR, mendelian randomization; VATV, visceral adipose tissue volume. | | | | | | |


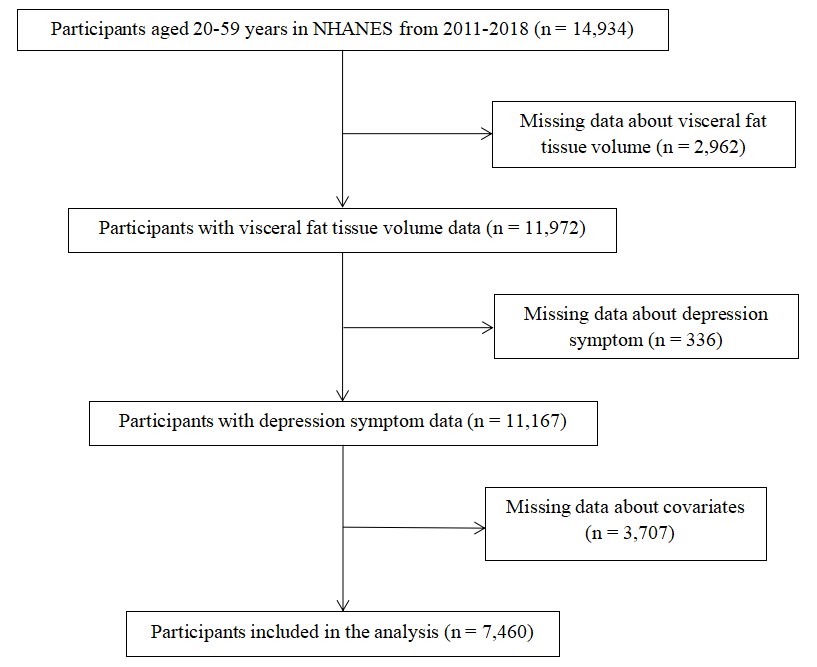


**Supplementary Figure 1.** Flowchart of sample selection in NHANES (2011-2018).


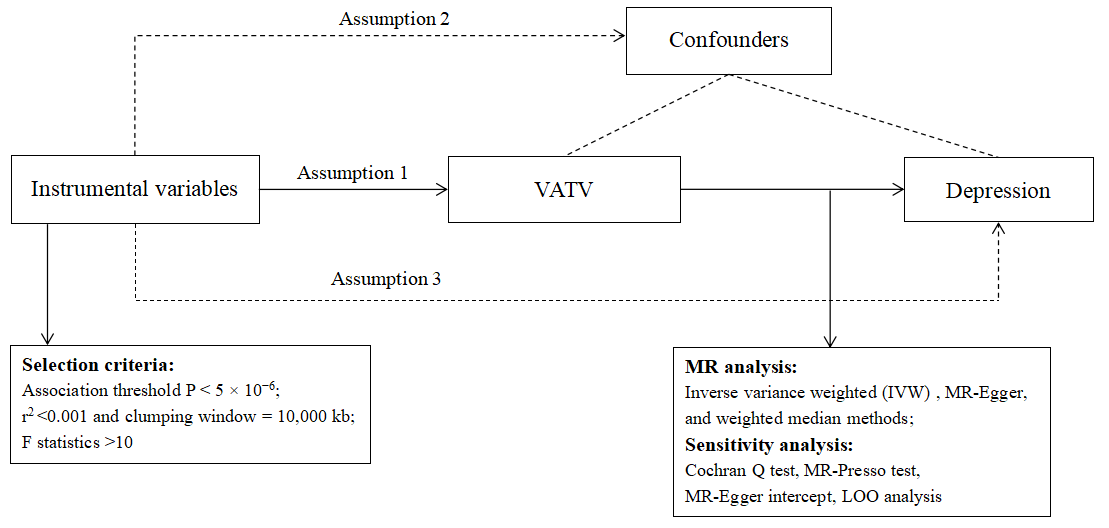


**Supplementary Figure 2.** Principles of MR and assumptions. Assumption 1: exposure is robustly associated with genetic variants; Assumption 2: confounders are not associated with genetic variants; Assumption 3: genetic variants should influence the outcomes only mediated by the exposure of interest. MR, Mendelian randomization; VATV, visceral adipose tissue volume.


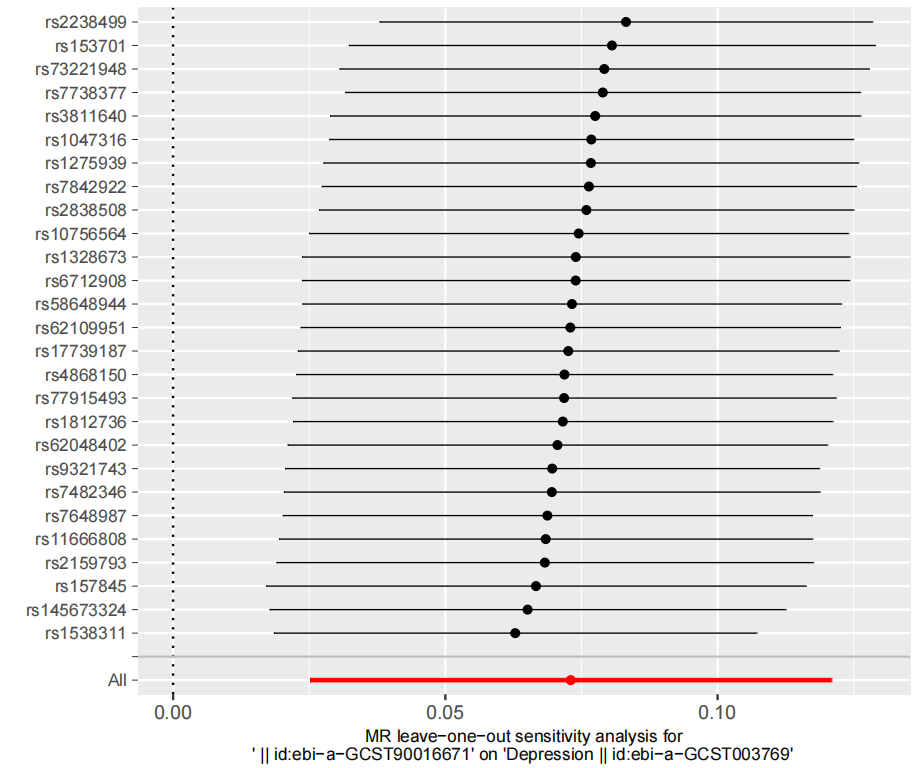


**Supplementary Figure 3.** MR leave-one-out sensitivity analysis for VATV on depression. MR, Mendelian randomization; VATV, visceral adipose tissue volume.


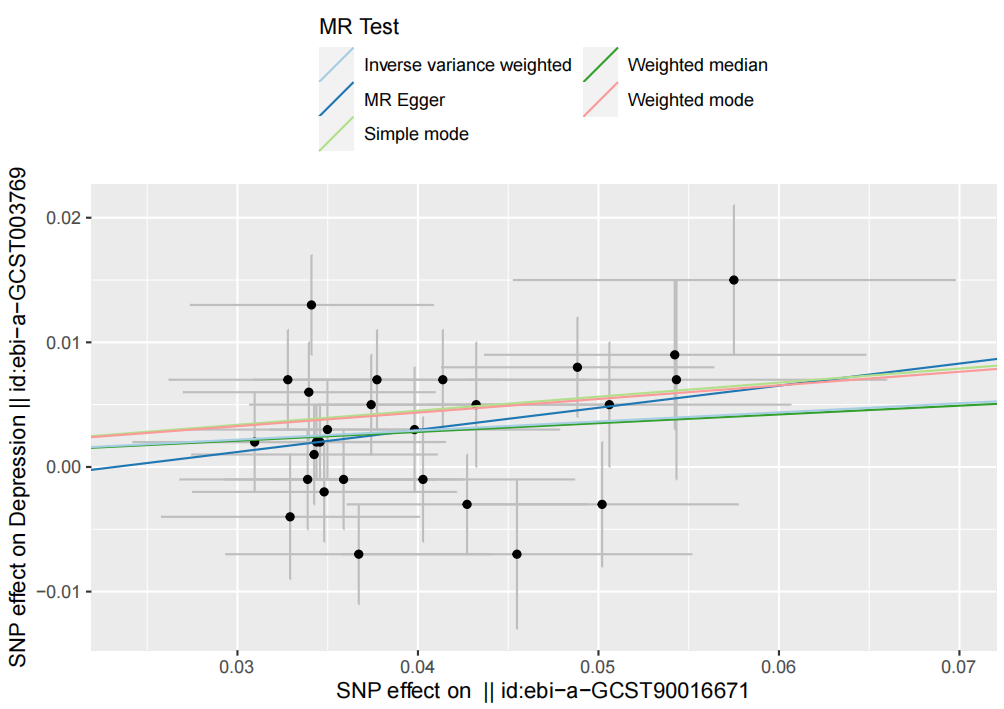


**Supplementary Figure 4.** SNP effect on depression.


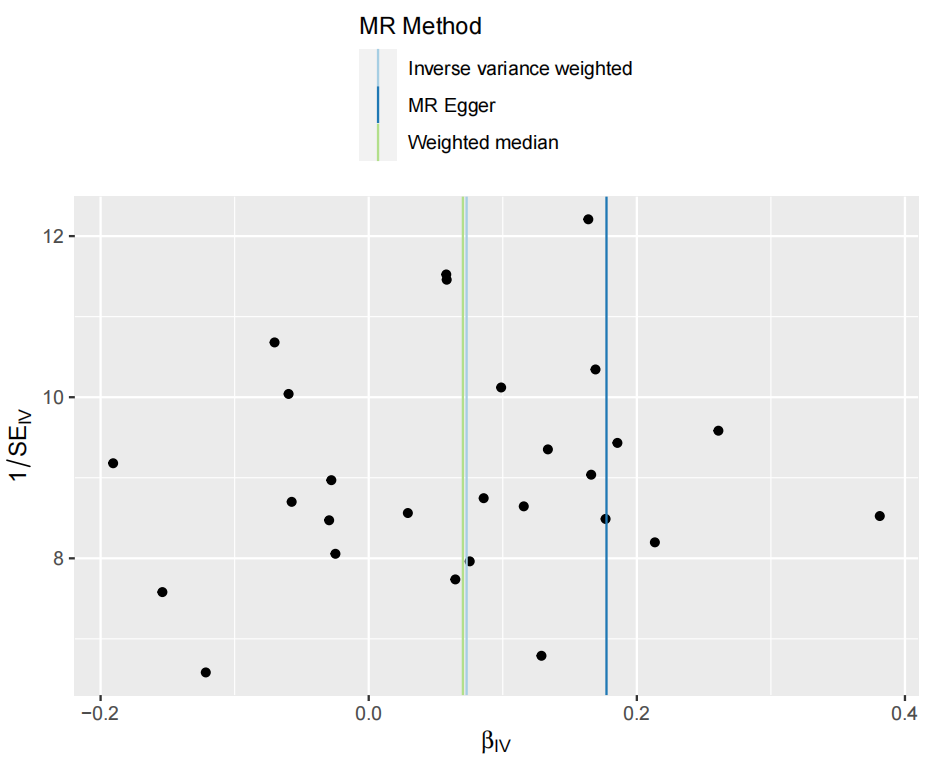


**Supplementary Figure 5.** Funnel plot of SNP effect on depression.
